# Functionalized Poly(*N*-isopropylacrylamide)-Based Microgels in Tumor Targeting and Drug Delivery

**DOI:** 10.3390/gels7040203

**Published:** 2021-11-08

**Authors:** Simona Campora, Reham Mohsen, Daniel Passaro, Howida Samir, Hesham Ashraf, Saif El-Din Al-Mofty, Ayman A. Diab, Ibrahim M. El-Sherbiny, Martin J. Snowden, Giulio Ghersi

**Affiliations:** 1 Department of Biological, Chemical and Pharmaceutical Sciences and Technologies (STEBICEF), University of Palermo, Viale delle Scienze, Ed. 16, 90128 Palermo, Italy; simona.campora@unipa.it (S.C.); daniel.passaro@abbvie.com (D.P.); 2 Abiel s.r.l, c/o Arca Incubatore di Imprese, University of Palermo, Viale delle Scienze, Ed. 16 (Floor-2), 90128 Palermo, Italy; 3 Faculty of Biotechnology, October University for Modern Sciences and Arts, Cairo 12451, Egypt; rmohsen@msa.edu.eg (R.M.); ho.samir@nu.edu.eg (H.S.); hesham.ashraf@msa.edu.eg (H.A.); adiab@msa.eun.eg (A.A.D.); 4 School of Science, University of Greenwich, Gillingham, Chatham, Kent, Canterbury ME4 4TB, UK; m.j.snowden@greenwich.ac; 5 Center of Materials Science, Zewail City of Science and Technology, 6th October City, Giza 12588, Egypt; s-saifel-din.el-mofty@zewailcity.edu.eg (S.E.-D.A.-M.); ielsherbiny@zewailcity.edu.eg (I.M.E.-S.)

**Keywords:** p(NIPAM)-co-5%AA microgels, folic acid, doxorubicin, cancer

## Abstract

Over the past several decades, the development of engineered small particles as targeted and drug delivery systems (TDDS) has received great attention thanks to the possibility to overcome the limitations of classical cancer chemotherapy, including targeting incapability, nonspecific action and, consequently, systemic toxicity. Thus, this research aims at using a novel design of Poly(*N*-isopropylacrylamide) p(NIPAM)-based microgels to specifically target cancer cells and avoid the healthy ones, which is expected to decrease or eliminate the side effects of chemotherapeutic drugs. Smart NIPAM-based microgels were functionalized with acrylic acid and coupled to folic acid (FA), targeting the folate receptors overexpressed by cancer cells and to the chemotherapeutic drug doxorubicin (Dox). The successful conjugation of FA and Dox was demonstrated by dynamic light scattering (DLS), Fourier-transform infrared (FTIR) spectroscopy, thermogravimetric analysis (TGA), UV-VIS analysis, and differential scanning calorimetry (DSC). Furthermore, viability assay performed on cancer and healthy breast cells, suggested the microgels’ biocompatibility and the cytotoxic effect of the conjugated drug. On the other hand, the specific tumor targeting of synthetized microgels was demonstrated by a co-cultured (healthy and cancer cells) assay monitored using confocal microscopy and flow cytometry. Results suggest successful targeting of cancer cells and drug release. These data support the use of pNIPAM-based microgels as good candidates as TDDS.

## 1. Introduction

Cancer is one of the leading causes of death in the world. In 2020, the world health organization stated that the number of deaths caused by cancer reached ten million deaths worldwide [data from WHO] [1]. One of the most commonly used therapies is chemotherapy, which is delivered systematically in a non-targeted manner [2]. Over the past several decades, the development of engineered nano- and micro-systems for targeted drug delivery have received great attention thanks to their possibility to overcome the limitations of classical cancer chemotherapy, including poor solubility, targeting incapability, nonspecific action and, consequently, systemic toxicity [3,4]. For instance, the anticancer drug doxorubicin (Dox) showed several adverse effects, such as myelosuppression, which is the decrease in the ability of the bone marrow to produce new blood cells, vomiting, and in extreme cases, it can lead to liver dysfunction and heart diseases. All these adverse effects are due to the apoptosis of healthy cells along with cancer cells as a result of untargeted drug administration [5]. Recently, scientists have developed targeted drug delivery systems using smart particles against cancer cells to reduce the side effects of chemotherapy [6].

Ligand-mediated targeting is based on the conjugation of engineering particles to specific targeting molecules including small molecules, carbohydrates, antibodies, or peptides in order to bind to specific receptors present on the surface of cancer cells [7,8]. For instance, the low molecular weight, low production cost, and ease of nano- and micro-particles systems conjugation make small molecules optimal candidates as potential targeting ligands. Folate receptors are known to be overexpressed almost 100–300 times more in cancer cells than normal ones; this is to increase the cancer cells’ uptake of folic acid (FA) used in different cellular metabolic pathways [9]. Accordingly, small particles can be conjugated with FA that binds specifically to folate receptors, to achieve targeted therapy [10].

Therefore, nanoparticles and microparticles have to be synthesized, engineered and optimized to raise the circulating half-life and to obtain a site-specific release of drugs at therapeutically optimal levels and dose regimes [11]. The composition, size, shape, surface properties, biocompatibility, and degradation profile should be carefully considered for the optimal design of the NPs for therapeutic purposes [12,13]. Depending on the aim and the particles nature, the drug can be encapsulated [14], conjugated by stacking interactions [15], or by chemical reactions [16,17] and the drug release can be induced in a stimuli-responsive way [18,19].

Among the different particle types, the nano- and micro-gels present many advantages, including high mechanical properties, stability, high water content, large flexible surface for the conjugation with a big amount of cargo protected in an aqueous environment, as well as biocompatibility [20]. They are constituted of polymer chains that form a matrix able to absorb and retain high quantity of aqueous solution (swelling capacity) [21,22].

In this contest, Poly(*N-iso*-propyl acrylamide)-*co*-Acrylic Acid (p(NIPAM)-co-5%AA) are smart polymeric microgels that change their physiochemical behavior in response to external stimuli such as temperature and pH change. These changes are instantaneous and reversible, as they return to their original status once the stimulus is removed [21,22]. As for other smart materials, p(NIPAM)-co-AA has been studied for different applications, such as tissue engineering scaffolds, cell culture supports, and bioseparation devices [23,24]. Smart particles can be used to reduce the adverse effects of the drug, increasing its efficiency, reducing the dosage, and consequently its cost [25]. The expected advantages of using p(NIPAM)-based particles have led researchers such as Guo et al. to suggest p(NIPAM)-co-AA as a choice for targeted cancer therapy since the pH of the microenvironment surrounding tumor cells is known to be more acidic than that surrounding healthy ones [26]. Moreover, p(NIPAM)-co-AA respond to acidic environment by contracting its size al-lowing ease of absorption, while in alkaline environment the p(NIPAM)-co-AA swells in size, allowing difficulty of absorption towards the cells [27,28].

In this research, a targeted drug delivery system for cancer cells was designed and developed through covalent bonding of p(NIPAM)-co-5%AA to FA, as the targeting agent, and to Doxorubicin as the anti-cancer drug, through 1-Ethyl-3-(3-dimethylaminopropyl)carbodiimide (EDC) and *N*-hydroxysuccinimide (NHS) coupling chemistry. The study was performed on HB2 (healthy breast cells) and MDA-MB 231 (breast cancer cells). In vitro characterization was used to evaluate the physicochemical behavior of the microgel particles through ultraviolet–visible (UV-Vis) spectroscopy, differential scanning calorimetry (DSC), and dynamic light scattering (DLS) to calculate the size distribution against temperature change. This is in addition to thermogravimetric analysis (TGA) and Fourier-transform infrared spectroscopy (FTIR) as confirmation of successful coupling reaction of EDC/NHS with each stage of folic acid conjugation and Dox conjugation. The cell biocompatibility of different concentrations of p (NIPAM)-co-5%AA, as well as p (NIPAM)-co-5%AA-co-FA and the cytotoxic effect of p (NIPAM)-co-5%AA-co-FA-co-Dox were tested. Finally, the specific tumor targeting experiments that test the suggested targeting behavior of the particles qualitatively and quantitatively were carried out. These are confocal microscopy and flow cytometry.

## 2. Results and Discussion

### 2.1. Synthesis of p(NIPAM)-co-5%AA Microgels and Conjugation with Folic Acid and Doxorubicin

**Figure 1:**
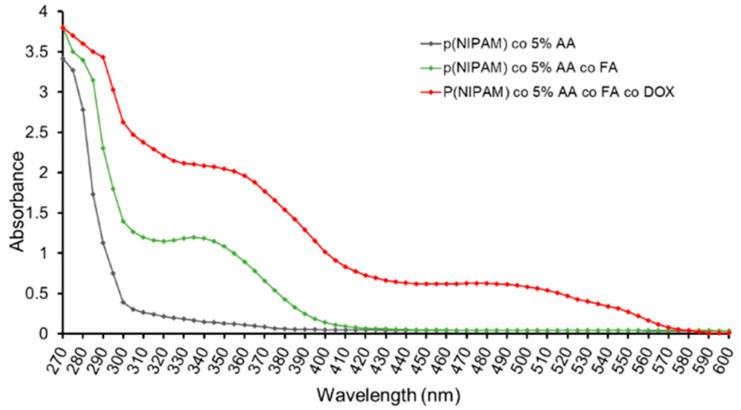
UV-VIS spectra of p(NIPAM)-co-5%AA, p(NIPAM)-co-5%AA-co-FA, and p(NIPAM)-co-5% AA-co-FA-co-Dox.

A sequential synthesis and conjugation processes were performed to generate microgel particles decorated with the targeting molecule folic acid and the anticancer drug doxorubicin. p(NIPAM)-co-5%AA were synthesized by Surfactant Free Emulsion Polymerisation (SFEP) technique as described in materials and methods to avoid toxic surfactant contamination [28,29]. Successively, EDC-NHS protocol was adopted to first bind folic acid to some of the acrylic acids of p(NIPAM)-co-5%AA microgels and then doxorubicin to the remaining acrylic acid residues. The success of the protocol was demonstrated by the UV-VIS analysis in which it was evident the characteristic peak of folic acid (340 mm) on p(NIPAM)-co-5%AA-co-FA and both folic acid and doxorubicin (485 nm) peaks on p(NIPAM)-co-5%AA-co-FA-co-Dox (Figure 1). The amount of folic acid and doxorubicin conjugated was calculated by spectrophotometric analysis using the standard calibration curves (Figures S1 and S2).

### 2.2. Size of Microgels

**Figure 2:**
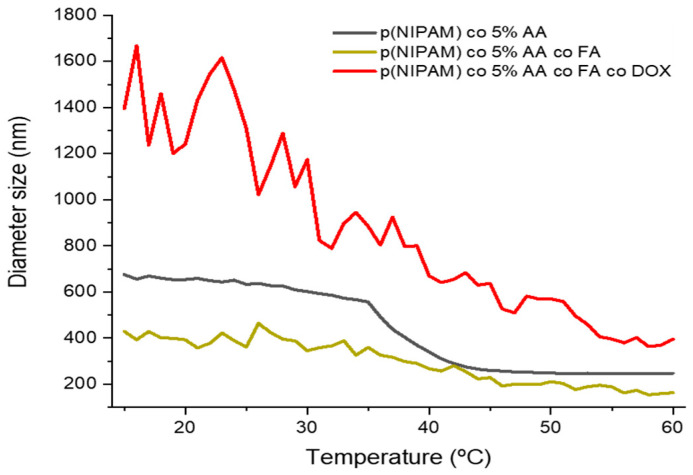
Size change of p(NIPAM)-based microgels against heating cycle temperature. The PDI for p(NIPAM)-co-5%AA, p(NIPAM)-co-5%AA-co-FA, and p(NIPAM)-co-5%AA-co-FA-co-Dox is 0.107, 0.482, and 0.531, respectively.

The effect of temperature change on the size of p(NIPAM)-co-5%AA, p(NIPAM)-co-5%AA-co-FA, and p(NIPAM)-co-5%AA-co-FA-co-DOX was studied by dynamic light scattering analysis (DLS) (Figure 2 and Figure S3). The size of the three microgel particles showed typical microgel behavior [30]. Below the VPTT (volume phase transition temperature) (34 ºC), the particles were swollen and configure a large size. At 34 °C (VPTT), the three microgels underwent a sharp decrease in size as the hydrogen bonds between the polymer particles and water molecules break due to energy gained under higher temperature [30,31], causing the polymer–polymer interactions to dominate. Hence, water molecules were expelled from microgel particles, causing the microgel to collapse and deswell [30,32].

At 15 °C, p(NIPAM)-co-5%AA had an average diameter of 701 nm while that of p(NIPAM)-co-5%AA-co-FA had an average diameter of 451 nm particle size. This was because FA, being a large molecule with several hydrophobic aromatic moieties, tended to decrease the hydrophilicity of the particle and decrease the hydrogen bonding with water molecules, hence it contained less water than that of the AA one. Further conjugating the particles with Dox molecules had increased the length of the hairy layers, hence causing the particle to increase in size at an average diameter of 1500 nm. Doxorubicin, being another bulky molecule with several hydrophilic groups, had helped the microgels to swell and reach the micro-scale.

Attaching FA to the microgel particles, the microgel’s VPTT was unaffected but the size of the microgel was reduced even further. In the case of p(NIPAM)-co-5%AA-co-FA-co-DOX, a rapid and sharp decrease in size was observed. At 50 ºC, particles of the three microgels p(NIPAM)-co-5%AA, p(NIPAM)-co-5%AA-co-FA, and p(NIPAM)-co-5%AA-co-FA-co-Dox were deswollen to an average size of 247, 177, and 433 nm, respectively. Moreover, calculating the deswelling degrees between the minimum and maximum temperature is rather challenging. This is because the size of p(NIPAM)-co-5%AA-co-FA, tends to fluctuate greatly from 300 to 504 nm, then dropping back again to 400. The decrease in size of p(NIPAM)-co-5%AA-co-FA in comparison to p(NIPAM)-co-5% AA is due to the decreased hydrophobicity of the particles because of the hydrophobic rings in the molecular structure of folic acid. The hydrophobic structure of the molecule decreases the hydrogen bonding between the particle and water and hence decreases the amount of water entrapped within the particles. After adding Dox with a complex structure and large molecules, the particle size tends to increase due to elongated hairy structures [30].

It is worth mentioning that the overall PDI (polydispersity index) of p(NIPAM)-co-5%AA was 0.057, which indicated the highly satisfactory consistency between particle size and distribution. Attaching FA molecules to the above-mentioned microgels decreased this consistency and increased the overall PDI to reach 0.503, which was fairly satisfactory. However, the conjugation of the bulky Dox molecules had increased the overall PDI to 0.833. The reason for this increase in PDI was the fact that Dox is a bulky molecule. When Dox is chemically conjugated to p(NIPAM)-co-5%AA-co-FA, it can either attach to FA moiety or to the unreacted AA, which gives the microgel versatility to have free end FA moiety on the surface of the microgel to target the folate receptor.

### 2.3. Electrophoretic Mobility

**Figure 3:**
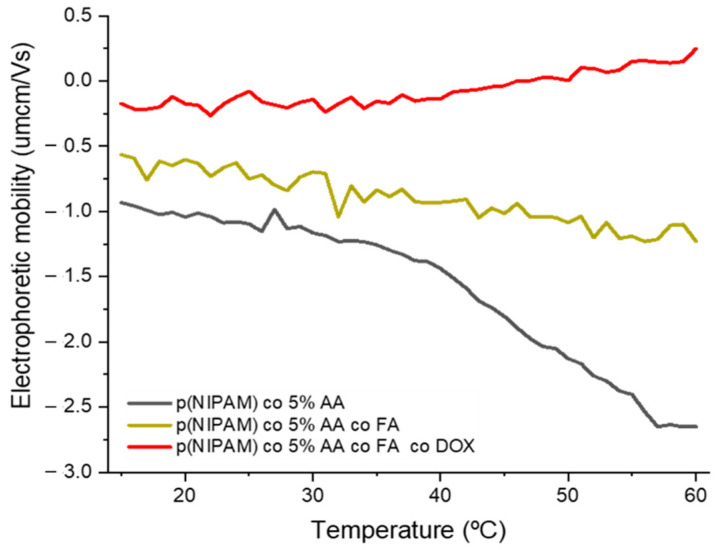
Electrophoretic mobility change of for p(NIPAM)-co-5%AA, p(NIPAM)-co-5%AA-co-FA, and p(NIPAM)-co-5%AA-co-FA-co-Dox versus temperature change (heating cycle).

Electrophoretic mobility (Em) of microgel particles is mainly affected by three factors: the size of microgels, solvent viscosity, and dielectric constant [33]. The latter two factors are needed to be kept at a minimum to measure the Em of microgel particles accurately across the temperature range, hence the usage of DI water as the dispersant [34]. The three microgels, p(NIPAM)-co-5%AA, p(NIPAM)-co-5%AA-co-FA, and p(NIPAM)-co-5%AA-co-FA-co-Dox, showed an increase in their magnitude of Em (|Em|) as the temperature increased from 15–60 ºC (Figure 3 and Figure S4).

At 15 °C, p(NIPAM)-co-5%AA had a negative electrophoretic mobility of average -0.946 µmcm/Vs. While that of p(NIPAM)-co-5%AA-co-FA average E_m_ is −0.401 µmcm/Vs, which showed that conjugating p(NIPAM)-co-5%AA to FA resulted in a decrease in its E_m._ p(NIPAM)-co-5%AA-co-FA-co-Dox had an average E_m_ of -0.0364 µmcm/Vs, this was due to the positive charge density of Dox, as well as, the bulky structure of the particle that causes the negative charges from the sulphate ions to be masked [33,35].

At 37 °C, the particle size dramatically decreased, which causes an increase in the surface charge density, hence an increase in electrophoretic mobility. In the case of p(NIPAM)-co-5%AA, the increase in electrophoretic mobility around VPTT was sharp. This was because the negative charges were exposed, while in case of p(NIPAM)-co-5%AA-co-FA and p(NIPAM)-co-5%AA-co-FA-co-Dox, it was suggested that the complex structure of the particle had masked some of the charges causing the increase in E_m_ to be steep.

### 2.4. Thermogravimetric Analysis (TGA)

**Figure 4:**
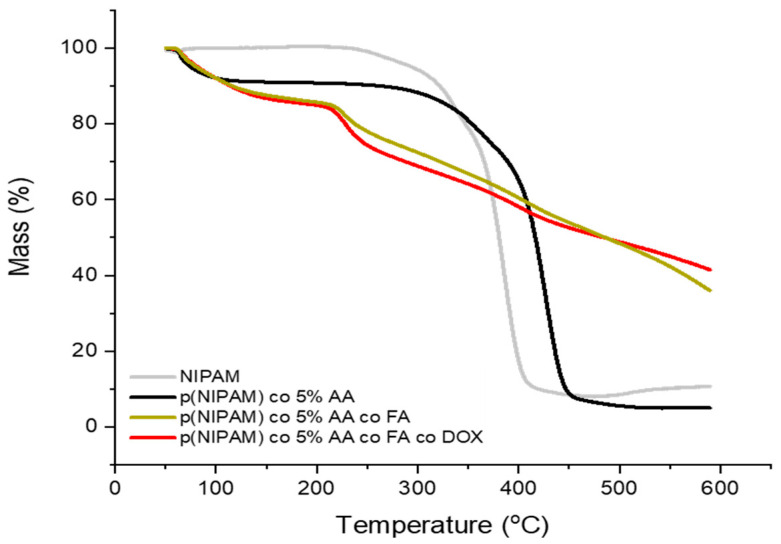
TGA curves of p(NIPAM), p(NIPAM)-co-5%AA, p(NIPAM)-co-5%AA-co-FA, and p(NIPAM)-co-5%AA-co-FA-co-Dox from ambient room temperature to 600 °C.

TGA (Thermogravimetric Analysis) analysis in Figure 4 shows the thermostability of microgel particles, in terms of mass percentage retained against temperature under ambient atmosphere. p(NIPAM) was thermally stable up till 250 °C where afterwards it started to decrease in mass. This was because the microgel gets burnt in the presence of oxygen until it reached a plateau at 400 °C and p(NIPAM) was turned to ashes (which is the remaining mass). p(NIPAM)-co-5%AA experienced a similar sigmoid curve as plain p(NIPAM), but showed higher thermal stability as it decreased in mass at 290 °C and reached a plateau at 440 °C.

p(NIPAM)-co-5%AA-co-FA, and p(NIPAM)-co-5%AA-co-FA-co-Dox exhibited similar behavior in thermal stability to one another. The steady decrease in mass over a wide range of temperatures indicates that FA led to an increase in thermal stability and slow decomposition for the p(NIPAM) microgels, this was due to the chemical conjugation of FA to p(NIPAM)-co-5%AA. FA is a thermal stable moiety and degrades slowly at high temperatures, and as such, FA sustained p(NIPAM)-co-5%AA-co-FA and p(NIPAM)-co-5%AA-co-FA-co-Dox microgels up to 40% of their masses at 600 °C [36]. It can then be concluded that FA had been chemically conjugated to p(NIPAM)-co-5%AA microgels due to the high thermal stability.

### 2.5. Differential Scanning Calorimetry (DSC)

**Figure 5:**
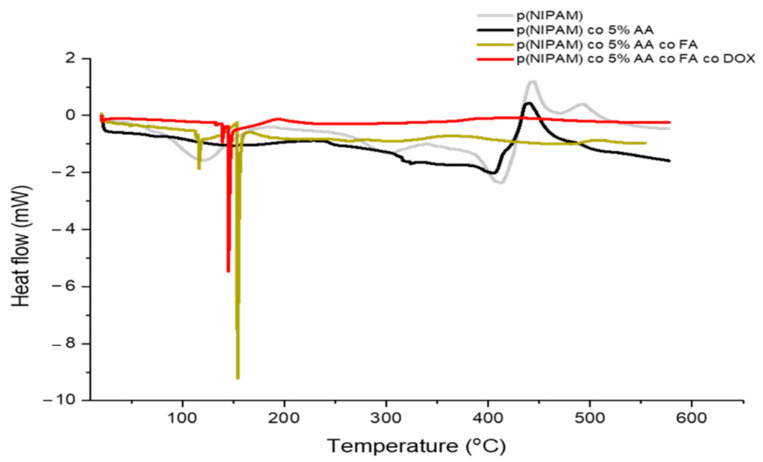
The DSC graph of p(NIPAM), p(NIPAM)-co-5%AA, p(NIPAM)-co-5%AA-co-FA, and p(NIPAM)-co-5%AA-co-FA-co-Dox under nitrogen atmosphere at a temperature range of rtp-600 °C.

The thermal behavior of p(NIPAM) and p(NIPAM)-co-5%AA undergoes two stages, these are melting of crystallization (micro-melting) and the melting point of the sample. Differential Scanning Calorimetry (DSC) showed that the first stage melting of crystallization occurs at 116 °C for p(NIPAM), while it occurred further in p(NIPAM)-co-5%AA at 153 °C. Furthermore, a series of endothermic peaks at 411 °C for p(NIPAM) and 404 °C for p(NIPAM)-co-5%AA indicating their melting points was registered. Further heating exhibited two-step exothermic peaks for p(NIPAM), but one for p(NIPAM)-co-5%AA.

p(NIPAM)-co-5%AA-co-FA and p(NIPAM)-co-5%AA-co-FA-co-Dox exhibited a lower crystallization melting point at an endothermic peak of 116 °C for p(NIPAM)-co-5%AA-co-FA and 131 °C for p(NIPAM)-co-5%AA-co-FA-co-Dox. Melting points of p(NIPAM)-co-5%AA-co-FA and p(NIPAM)-co-5%AA-co-FA-co-Dox were 154 and 145 °C, respectively and did not exhibit any exothermic peaks like the other two microgels (Figure 5). This indicates that the change in thermal behavior in p(NIPAM)-co-5%AA-co-FA and p(NIPAM)-co-5%AA-co-FA-co-Dox was due to the moieties that were chemically conjugated to p(NIPAM)-co-5%AA. Moreover, the existence of only one melting point in each p(NIPAM)-co-5%AA-co-FA and p(NIPAM)-co-5%AA-co-FA-co-Dox indicated the purity of the sample and that nothing else was co-existing with these microgels.

### 2.6. Fourier Transform Infra-Red Spectroscopy (FTIR)

**Figure 6:**
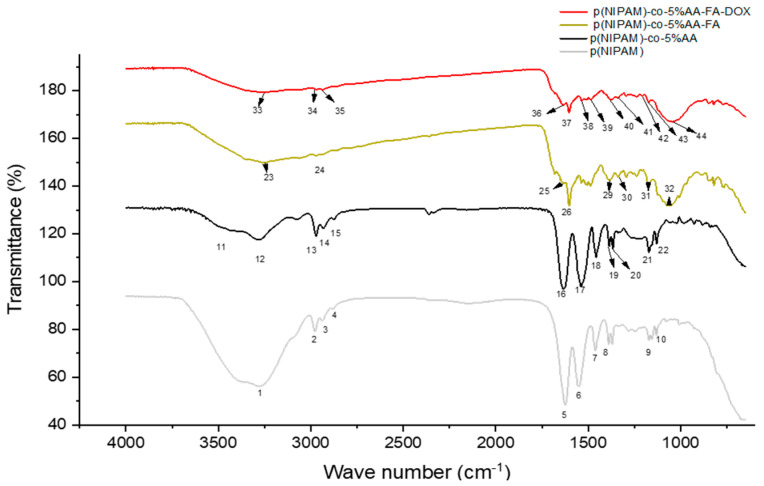
FTIR of p(NIPAM), p(NIPAM)-co-5%AA, p(NIPAM)-co-5%AA-co-FA, and p(NIPAM)-co-5%AA-co-FA-co-Dox showing the peaks that signify the chemical conjugates of each moiety.

The FTIR (Fourier Transform Infra-Red Spectroscopy) spectra of the three microgels are shown in Figure 6, while the peaks and their assignments are mentioned in Table 1. The FTIR of p(NIPAM)-co-5%AA showed a peak at 3417 cm^−1^ of the hydroxyl group of the carboxylic acid and the C=O in the carboxylic acid group. The sulphate ions were expressed at 1130 cm^−1^. It was also worth noting that some peaks that were available in p(NIPAM) were shifted in p(NIPAM)-co-5%AA, these include 3283, 2972, 2933, 2876, 1632, 1538, 1457, and 1386 cm^−1^.

p(NIPAM)-co-5%AA-co-FA had additional functional groups due to the presence of FA, such as the aromatic ring in FA, the aryl stretch 1603 cm^−1^ and aryl C=C at 1487 cm^−1^ and a heterocyclic ring containing secondary amine 1339 cm^−1^.

Finally, p(NIPAM)-co-5%AA-co-FA-co-Dox had few additional functional groups that are expressed exclusively for Dox in its spectrum including the 13C-H and COH stretch of Dox occurring at 1377 and 1209 cm^−1^, which are very unique to Dox [37].

The FTIR results of p(NIPAM)-co-5%AA-co-FA and p(NIPAM)-co-5%AA-co-FA-co-Dox, and the shift in wavenumbers that were observed in the spectra (Figure 5 and Table 1) were confirmatory results that the moieties were chemically conjugated and that FA and Dox were not ionically interacting with the p(NIPAM)-co-5%AA microgels, as it would have diffused out through the dialysis step.

### 2.7. Biocompatibility of p(NIPAM)-co-5%AA and p(NIPAM)-co-5%AA-co-FA Microsystems

**Figure 7:**
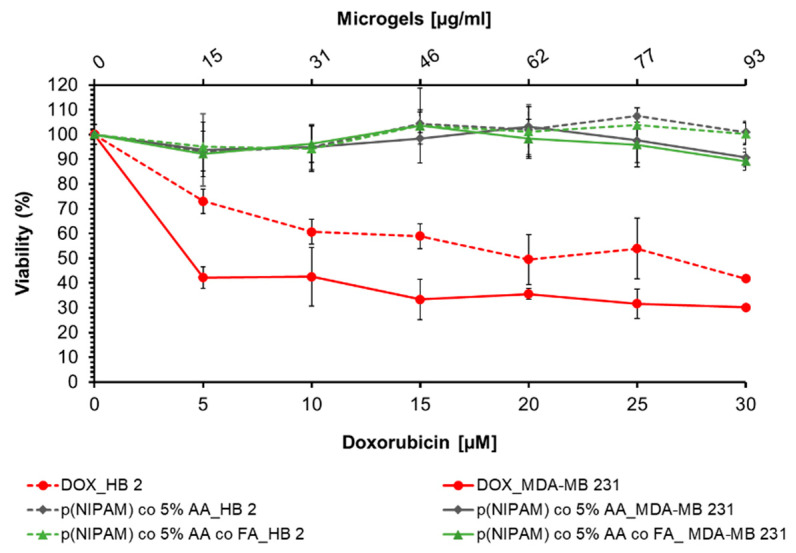
Viability assay on HB2 and MDA-MB 231 cells incubated for 24 h with p(NIPAM)-co-5%AA, p(NIPAM)-co-5%AA-co-FA (5; 10; 15; 20; 25; 30 µg/mL). Cells treated with doxorubicin were used as positive control, while untreated cells were used as negative control.

Viability assay was initially performed on cells treated with microgels without any anticancer drug conjugated, used as a control, in order to verify their biocompatibility. Therefore, CCK-8 (Cell counting kit-8) assay was performed on normal (HB2) and tumor (MDA-MB 231) cells treated for 24h with different concentrations (15, 31, 46, 62, 77, and 93 µg/mL) of p(NIPAM)-co-5%AA or p(NIPAM)-co-5%AA-co-FA microgels. Cells treated with doxorubicin (5, 10. 15, 20, 25, and 30 µM) were used as positive control. As reported in Figure 7, microgel particles alone or conjugated with folic acid do not alter the cell viability of both normal and tumor cells (viability of around 100%), also if used at high concentration (92.88 µg/mL). Furthermore, cell viability was also maintained at a higher concentration (100 µg/mL) of p(NIPA)-co-5%AA until 48 h of treatment and the cell morphology was not altered as suggested by acridine orange assay (Figures S5 and S6).

### 2.8. Qualitative Uptake of p(NIPAM)-co-5%AA-co-FA-co-Dox

Once established the biocompatibility of microgels, cell internalization uptake was initially investigated by fluorescence microscopy by incubating MDA-MB 231 cells with a fluorescence variant of microgel particles over time, as reported in supporting information (Figures S7 and S8). The green fluorescence relative to microgels appeared localized in specific areas, probably corresponding to the Golgi apparatus or the endoplasmic reticulum after 1 h of incubation (Figures S7c–c’’ and S8).

**Figure 8:**
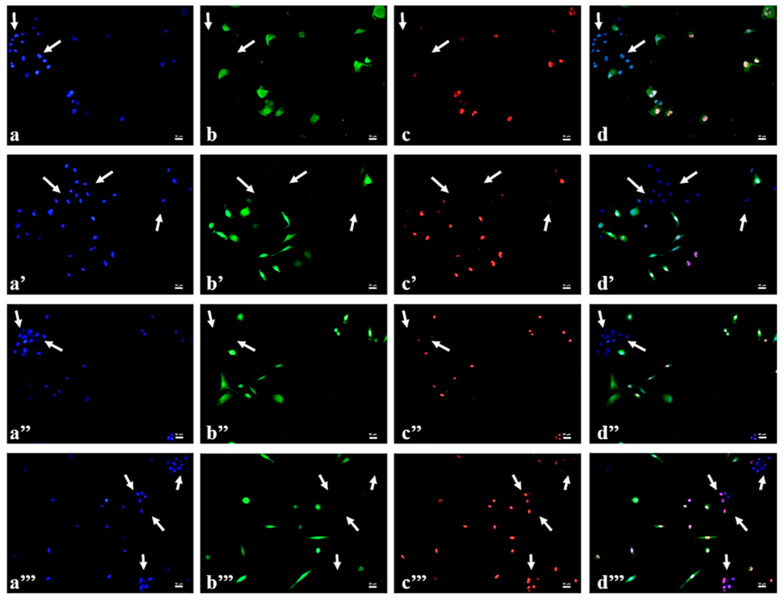
Fluorescence images of co-culture of HB2 (blue) and MDA-MB231 (blue and green) cells incubated with p(NIPAM)-co-5%AA-co-FA-co-Dox (10 µM) (red) for 30 min (**a**–**d**); 1 h (**a’**–**d’**); 2 h (**a’’**–**d’’**), and 4 h (**a’’’**–**d’’’**). Blue: nuclei (DAPI); Green: MDA-MB 231 cells (CellTrace CFSE); Red: doxorubicin of p(NIPAM)-co-5%AA-co-FA-co-Dox microgels. Magnification 20×. Scale bar: 50 µm.

Fluorescence microscopy was also adopted to investigate the specific tumor targeting of microgels functionalized with folic acid. The folate receptor (FR) is overexpressed in the majority of human tumors, like breast, and, in particular, MDA-MB231 cells produce high FR concentration [40]. Therefore, a co-culture experiment was performed by seeding HB2 and green-labelled MDA-MB 231 cells together and incubating them with p(NIPAM)-co-5%AA-co-FA-co-Dox microgels or doxorubicin alone as control (identified by the doxorubicin red auto-fluorescence, Figure 8c–c’’’ and Figure S9c–c’’’). Nuclei of both cells were stained with DAPI (blue fluorescence, Figure 8a–a’’’ and Figure S9a–a’’’) so that HB2 healthy cells were identified by blue fluorescence alone, while MDA-MB 231 tumor cells were individuated by both blue and green fluorescence. Following the microgel particles cellular uptake over time, it was evident the presence of the red fluorescence (corresponding to doxorubicin conjugated to the particles) exclusively in tumor cells already at the shortest incubation time (30 min, Figure 8a–d) and more and more at the following incubation times (1, 2, and 4 h, Figure 8a’–d’,a’’–d’’,a’’’–d’’’). On the other hand, red fluorescence was totally absent in correspondence of HB2 cells (white arrows in Figure 8d–d’’), suggesting a specific tumor targeting of p(NIPAM)-co-5%AA-co-FA-co-Dox microparticles. The red fluorescence, relative to doxorubicin, began to appear in HB-2 cytoplasm in 4 h, as expected by static in vitro system. On the contrary, the soluble form of the doxorubicin was inside both normal and tumor cells already after 30 min of treatment, suggesting that microparticles, conjugated with folic acid, were responsible for the selectively for cancer cells (Figure S9). The co-localization of the blue (nuclei) and the red (doxorubicin) fluorescence in tumor cells (Figure 8) suggested that the drug was released from the microgels and entered into the nuclei, which can intercalate into the DNA causing cell death. On the other hand, microgels fluorescence signal was always localized in the cytoplasm (Figures S7 and S8).

### 2.9. Quantitative Uptake Study

**Figure 9:**
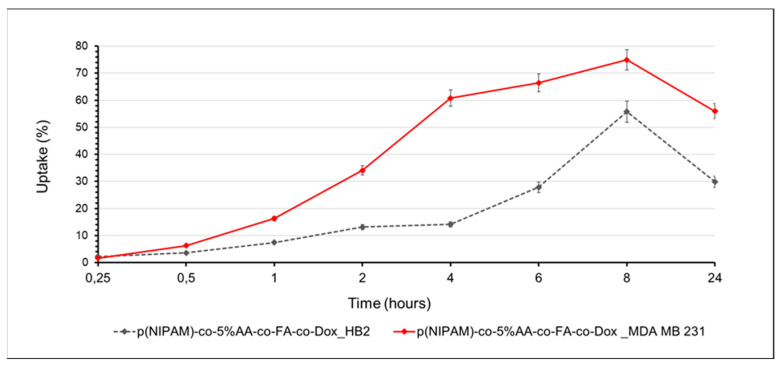
Uptake percentage of p(NIPAM)-co-5%AA-co-FA-co-Dox (doxorubicin conjugated concentration of 20 µM) by HB2 and MDA-MB 321 cells during different incubation times.

Differential microgel particles cellular uptake between normal and tumor cells was furthermore investigated by the quantitative flow cytometric analysis, following the red autofluorescence of conjugated doxorubicin (Figure 9 and Figure S10). Initially (30 min), there were no significant differences in p(NIPAM)-co-5%AA-co-FA-co-Dox internalization between HB2 (breast healthy cells) and MDA-MB-231 (breast cancer cells). After 1 h of incubation, the uptake gap started to increase, suggesting a specific tumor targeting due to the conjugated folic acid, reaching the maximum value after 4 h of treatment: the microgels internalization in tumor cells was 60% against the 14% of internalization into normal cells.

After 6 and 8 h, the amount of p(NIPAM)-co-5%AA-co-FA-co-Dox inside MDA-MB 231 cells increased slowly (66 and 75%, respectively), suggesting the reaching of the maximum cell internalization. By contrast, it increased inside normal cells, as expected for longer incubation time in a static in vitro system. In summary, the particle uptake ratio at 0.5, 1, 2, 4, 6, 8, and 24 h was 1.7, 2.2, 2.6, 4.3, 2.3, 1.3, and 1.8, respectively. This showed that the maximum difference in particle uptake was a ratio of 4.3 after 4 h of incubation, suggesting that p(NIPAM)-co-5%AA-co-FA-co-Dox targeted MDA-MB 321 cancer cells due to the recognition between folate and its receptor. On the contrary, in HB2 healthy cells, which present lower FR expression, the microparticles uptake was time-delayed, suggesting again a specific particles tumor targeting. The decrease registered at 24 h of incubation for both normal and tumor cells (30% and 56%, respectively) was correlated to the death of cells that initially had internalized particles.

### 2.10. Cytotoxicity Assay

The cytotoxic effect of doxorubicin conjugated to microparticles was evaluated on normal HB 2 and MDA-MB 231 tumor cells by a viability assay.

The selected doxorubicin concentrations corresponded exactly to the amount of drug conjugated to microgels analyzed in biocompatibility assay (Figure 7): 5, 10, 15, 20, 25, and 30 µM of the drug to 15, 31, 46, 62, 77, and 93 µg/mL of microgels, respectively.

**Figure 10:**
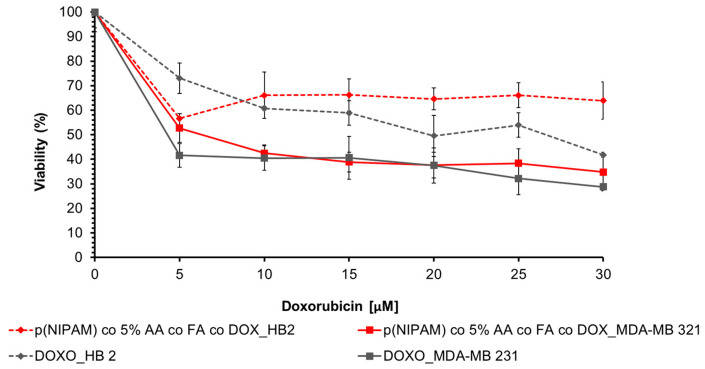
Cell viability assay on HB2 (normal) and MDA-MB 231 (cancer) cells incubated for 24 h with different concentrations of doxorubicin conjugated with p(NIPAM)-co-5%AA-co-FA-co-Dox. Cells incubated with the equivalent concentrations of doxorubicin were used as positive control.

As shown in Figure 10, 5 µM of the drug conjugated to p(NIPAM)-co-5%AA-co-FA-co-Dox induces cell mortality on MDA-MB 231 cells (48% of mortality) and the viability decreases in a concentration-dependent way, reaching the maximum efficiency at 15 µM, so that, at higher concentration, the plateau state was registered (around 37% at 20, 25, and 30 µM of Dox). These data suggest that conjugation protocol does not alter the structure and functionality of conjugated drug and, furthermore, that microsystems, can release the drug inside cells. On the contrary, the viability of healthy cells after incubation was around 66% for all the drug concentrations used, confirming again the specific targeting of p(NIPAM)-co-5%AA-co-FA-co-Dox to tumor cells. The small mortality of 33% registered in this case was due to the long treatment time in a static system (24 h). Doxorubicin alone was used as a positive control.

The differences in toxicity among different cell lines and microgels was probably due to the specific targeting of microgels to tumor cells, recognizing the folate receptor overexpressed by MDA-MB 231 cells. This brought a diverse cell internalization between tumor and normal cells as suggested by flow cytometry analysis, and therefore, to a distinct cytotoxic effect. It is worth mentioning that the biocompatibility of p(NIPAM) was previously tested by Mohsen et al. [41] when it showed cell viability over 90% at concentrations up to 3 mg/mL.

## 3. Conclusions

Although in the last years, cancer research has seen significant progress in the understanding, diagnosis, treatment, and prevention, low selectivity of the chemotherapeutic agents and consequently high side effects often occur. In this context, a novel drug delivery system that aims to specifically target cancer cells was designed and synthesized. Based on the tumor characteristic, p(NIPAM)-co-5%AA microgel particles were covalently conjugated to folic acid that is overexpressed in the majority of tumor cells (targeting agent) and to the anti-cancer drug Doxorubicin through EDC/NHS coupling reaction. The advantage of covalently tethering DOX, rather than loading it by self-assembly, is that the amount of DOX conjugated to the microgel is taken up almost completely. While the other self-assembly systems have either low entrapment efficiency (in case of synthetic polymers), or are not feasible to scale up (such as micelles) [42,43]. Moreover, tethering the DOX and conjugating it with a targeting moiety, ensures that DOX targets only cancer cells and shall be intracellularly released upon degradation of the microgel particles by relevant enzymes. Unlike other self-assembly systems, the DOX can be released in the bloodstream. Accordingly, it is suggested that calculating the needed doses of covalently tethered Dox can be easier and more accurate than a physically entrapped one.

The new delivery system was then characterized and tested for targeting ability and capability to release the conjugated drug inside cells.

Several characterization studies were carried out, including UV-Vis analysis, DLS, TGA, DSC, and FTIR to demonstrate the successful conjugation of FA and Dox to p(NIPAM)-co-5%AA microgel and that the new microgels retain microgel behavior [44].

The appearance of the typical FA and Doxo peaks in UV-VIS analysis (Figure 1) and the variation in size (DLS analysis, Figure 2) demonstrated a variety of microgel composition due to FA and Doxo conjugation. These data were confirmed by not only the variation of TGA curves (Figure 4), but also by the alteration of the DSC profiles of the microgels (Figure 5), shifting both the melting point and the crystallization melting point; furthermore, any exothermic peaks (that are present in p(NIPA) and p(NIPAM)-co-5%AA) were not registered. At the same time, also the FTIR profiles changed probably due to the different functional groups of the folic acid and doxorubicin. Taken together, these data confirmed the success of the conjugation, as demonstrated also by cytotoxic assay performed on normal and tumor cells (Figure 10) and the targeting studies (Figure 8 and Figure 9).

The uptake and localization studies of p(NIPAM)-co-5%AA-co-FA-co-Dox were performed using flow cytometry and fluorescence microscopy, while viability assay was carried out to investigate the cytotoxicity of the drug conjugated to developed microgels. Co-culture experiment demonstrated the drug release and the specific targeting of the microcomplex exclusively to the tumor cells by an active targeting that probably could be increased in vivo by a passive targeting based on the enhanced permeability and retention effect (EPR effect). Besides, viability assay results show higher cell viability for healthy cells incubated with p(NIPAM)-co-5%AA-co-FA-co-Dox than the cancer ones. Also, it is shown that at higher concentrations (25 µm and above), healthy cells were more viable when incubated with p(NIPAM)-co-5%AA-co-FA-co-Dox than when incubated with soluble form Dox. Therefore, these data suggest that p(NIPAM)-co-5%AA-co-FA-co-Dox are good candidates as delivery systems to increase the specific tumor targeting probably reducing general side effects, even if more in vivo studies need to be carried out.

## 4. Materials and Methods

### 4.1. Synthesis of p(NIPAM)-co-5%AA

A Surfactant Free Emulsion Polymerisation (SFEP) technique was used for the preparation of p(NIPAM)-co-5%AA as described previously and in accordance with literature [27,28,29,41]. Briefly, a three-neck lid was then fitted to the reaction vessel, which was placed onto a hot plate stirrer and heated to 70 °C with continuous stirring under N2 atmosphere. Potassium persulphate initiator (0.5 g) was dissolved in 800 mL of distilled water. The crosslinker *N*,*N*′-methylenebisacrylamide 99% (0.5 g) (BS, Sigma Aldrich, Gillingham, UK), *N*-isopropylacrylamide (NIPAM, Sigma Aldrich, Gillingham, UK) 97% monomer (4.75 g) and acrylic acid (AA, Sigma Aldrich, Gillingham, UK) co-monomer (0.25 g) were dissolved in 200 mL of distilled water while stirring gently with a magnetic stirrer. After all the reagents were dissolved, they were transferred into the reaction vessel containing the initiator. The reaction was run for 6 h with constant stirring and under nitrogen. After 6 h, the microgel dispersion was allowed to cool down to room temperature, then dialyzed (MW cut-off was 12–14,000 kDa) in fresh distilled water for 7 days.

### 4.2. Conjugation of p(NIPAM)-co-5%AA with Folic Acid

Folic acid (FA, Sigma Aldrich, Milano, Italy) was conjugated with p(NIPAM)-co-5%AA microgel particles by EDC/NHS protocol [45]. Briefly, p(NIPAM)-co-5%AA micorgels were suspended in 2-(*N*-morpholino) ethanesulfonic acid (MES, Sigma Aldrich, Milano, Milano, Italy) buffer solution (0.1 M, pH 5 with NaOH) at the final concentration of 5 mg/mL and sonicated for 20 min on ice bath in order to homogenize the solution. 1-ethyl-3-(3-dimethylaminopropyl) carbodiimide hydrochloride (EDC, Sigma Aldrich, Milano, Italy) was added 10 times more than NPs (*w/w*), mixed by vortex, and then *N*-hydroxysulfosuccinimide (Sulfo-NHS, Sigma Aldrich, Milano, Italy) powder was put (NPs/SulfoNHS = 4.5 *w/w*) [46,47]. The solution was then left for 30 min in agitation at room temperature and FA was added 10 times more than NPs (*w/w*) and mixed by a vortex. The solution of p(NIPAM)-co-5%AA and FA was then diluted with complete Phosphate Buffered Saline (PBS, Sigma-Aldrich, Milano, Italy) to reach a final NPs concentration of 1 mg/mL, the pH was adjusted to 7 using sodium bicarbonate and the solution was left for 2 h in agitation at room temperature.

The microparticles suspension was sonicated for 20 min at 37 °C and dialyzed to get rid of the unconjugated folic acid using a nitrocellulose tube (100 kDa cut-off). The dialysis buffer (distilled H_2_O) was changed twice a day for one week. Samples were sterilized by filtering with 0.22 µm filter and analyzed by spectrophotometric analysis [microplate reader DU-730 Life Science spectrophotometer (Beckman Coulter, Milano, Italy)] at 340 nm in order to determine the amount of folic acid conjugated to the microgel particles using a calibration curve (0.05; 0.10; 0.15; 0.20; 0.25; 0.30; 0.35; 0.40; 0.45; 0.50 µg/mL).

### 4.3. Conjugation of p(NIPAM)-co-5%AA-co-FA with Doxorubicin

After the freeze-drying process, p(NIPAM)-co-5%AA-co-FA were solubilized (1 mg/mL) on MES Buffer (0.1 M, pH 5 with NaOH) and sonicated on an ice bath for 20 min. EDC (10 times more than NPs *w/w*) and Sulfo-NHS (NPs/SulfoNHS = 4.5 *w/w*) were then added to the microparticles solution and mixed well by vortex and left in agitation at room temperature for 30 min. Doxorubicin (Dox, Sigma-Aldrich, Milano, Italy) powder was added to the solution (NPs/Dox = 1.2 *w/w*) and the final pH was adjusted to 7 using sodium bicarbonate. After 2.5 h of agitation at room temperature, the solution was sonicated for 20 min at 37 °C and put in a nylon membrane dialysis tube (14 KDa cut-off) in order to get rid of the unconjugated Dox. The dialysis buffer (distilled H_2_O) was changed twice a day for one week. Spectrophotometric analysis [microplate reader DU-730 Life Science spectrophotometer (Beckman Coulter, Milano, Italy)] was then performed for the p(NIPAM)-co-5%AA-co-FA-co-Dox solution at 485 nm to determine the amount of Dox conjugated to the microgel particles using a standard curve (5; 10; 20; 40; 60; 80; 100 µM).

### 4.4. Dynamic light Scattering (DLS) and Electrophoretic Mobility

p(NIPAM)-co-5%AA, p(NIPAM)-co-5%AA-co-FA, and p(NIPAM)-co-5%AA-co-FA-co-Dox were suspended in distilled water by 0.5% (*w/v*) using distilled water in a ratio of 1:2. The DLS software was programmed to measure the size [Zetasizer NS series (Malvern, Gillingham, UK)] and electrophoretic mobility in triplicates from 15 to 60 °C with a heating and cooling cycle.

### 4.5. Thermogravimetric Analysis (TGA)

Freeze-dried p(NIPAM), p(NIPAM)-co-5%AA, p(NIPAM)-co-5%AA-co-FA, and p(NIPAM)-co-5%AA-co-FA-co-Dox were weighed on platinum pans by the instrument [TGA Q50 (TA instruments, New Castle, DE, USA]. The system was heated under ambient air from room temperature to 600 °C at 10 °C/min.

### 4.6. Differential Scanning Calorimetry (DSC)

Known masses of freeze-dried p(NIPAM), p(NIPAM)-co-5%AA, p(NIPAM)-co-5%AA-co-FA, and p(NIPAM)-co-5%AA-co-FA-co-Dox were placed in Tzero aluminum pans and placed on the heater unit. The empty pan is placed in the reference heating unit and the system is heated from room temperature to 600 °C at 10 °C/min under nitrogen purge of 50 mL/min. [DSC Q20 (TA instruments, USA)].

### 4.7. Fourier-Transform Infrared Spectroscopy (FTIR)

The suspensions of p(NIPAM), p(NIPAM)-co-5%AA, p(NIPAM)-co-5%AA-co-FA and p(NIPAM)-co-5%AA-co-FA-co-Dox were freeze dried. The powders obtained were placed directly on diamond iTR of FTIR spectroscopy from 600 to 4000 cm^−1^ [FTIR Nicolet iS20 (thermoscientfic, Tewksbury, MA, USA)].

### 4.8. UV–Visible Spectra

UV–Visible spectra of p(NIPAM)-co-5%AA, p(NIPAM)-co-5%AA-co-FA, and p(NIPAM)-co-5%AA-co-FA-co-Dox were obtained using the range 270–600 nm at 5 nm increments, using 200 µL of each sample solution in 96 well plate (Synergy™ HT Multidetection microplate reader spectrophotometer (BioTek, Milano, Italy).

### 4.9. Cell Culture of HB2 and MDA-MB 231

MDA-MB 231 human breast cancer cells were grown in Dulbecco’s Modified Eagle’s Medium (DMEM, Sigma-Aldrich, Milano, Italy) high glucose (HG-DMEM) with 10% (*v/v*) Fetal bovine serum (FBS, Euroclone, Celbar, Pero (MI) Italy), 2 mM L-Glutamine (Euroclone, Celbar, Pero (MI) Italy), 100 units per mL penicillin G (Euroclone, Celbar, Pero (MI) Italy), 100 mg mL^−1^ streptomycin, while HB2 human mammary epithelial cells were grown in DMEM low glucose (LG-DMEM) with 10% (*v/v*) FBS, 4 mM L-Glutamine, 100 units per mL penicillin G, 100 mg mL^−1^ streptomycin, 5 mg mL^−1^ hydrocortisone (Sigma-Aldrich, Milano, Italy), and 10 µg mL^−1^ bovine insulin (Sigma-Aldrich, Milano, Italy). All cells were cultivated at 37 °C, in a humidified atmosphere of 5% CO_2_ and maintained in sterile conditions.

### 4.10. Viability of Cells Treated with Microgels

Viability assay was performed on MDA-MB 231 or HB2 cells incubated with p(NIPAM)-co-5%AA or p(NIPAM)-co-5%AA-co-FA microgel particles (Biocompatible assay) or with p(NIPAM)-co-5%AA-co-FA-co-Dox (Cytotoxic assay). Cells were seeded on 96-well plates at the density of 1 × 10^4^ cells/well and grown in the opportune medium at 37 °C for 24 h. Therefore, cells were treated with p(NIPAM)-co-5%AA or p(NIPAM)-co-5%AA-co-FA (15, 31, 46, 62, 77 and 93 µg/mL) or p(NIPAM)-co-5%AA-co-FA-co-Dox (5; 10; 15; 20; 25; 30 µM of conjugated drug) for 24 h and cell viability was detected by using Cell Counting Kit-8 (CCK-8, Sigma-Aldrich). In particular, water-soluble tetrazolium salt (WST-8) was added to each sample (1:10 dilution in complete medium) and incubated at 37 °C for 2 h to allow for its reduction by mitochondrial dehydrogenases of the living cells into soluble formazan dye that is directly proportional to the number of living cells. Spectrophotometric analysis [microplate reader DU-730 Life Science spectrophotometer (Beckman Coulter, Milano, Italy)] at 450 nm was then performed to determine the percentage of viable cells relative to the negative control (untreated cells). Cells treated with Doxorubicin were considered as a positive control.

### 4.11. Specific Targeting Cell Uptake

MDA-MB 231 cells (10^5^ cells per mL) were harvested by centrifugation and the cell pellet was incubated with 25 mM Molecular Probe CellTrace CFSE fluorescent stain (CellTrace CFSE Cell Proliferation Kit, Life Technologies, Italy) for 30 min at 37 °C.

For co-culture preparation, pre-labelled MDA-MB 231 and unlabeled HB2 cells were mixed (ratio 1:1) and seeded with a density of 8 × 10^4^ cells per well into 12-well plates containing sterile coverslips in complete LG-DMEM for grown 24 h at 37 °C.

In sterile conditions, cells were incubated with 10 µM of p(NIPAM)-co-5%AA-co-FA-co-Dox microgels for 15 min, 30 min, 1 h, 2 h, and 4 h. At the end of each incubation time, the cells were washed with PBS and then fixed with 3.7% formaldehyde (in PBS) for 5 min at room temperature, followed by three washes with PBS. Nuclei were stained in the dark with DAPI solution (dilution of 1:10,000 in water) for 15 min at room temperature. Samples were analyzed by fluorescence microscopy (Leica, Buccinasco (MI), Italy) and confocal microscope (FLUOVIEW FV10i-LIV, Olympus, Italy).

### 4.12. Quantitative Uptake by Flow Cytometry

MDA-MB 321 and HB2 cells were grown in 6 well plates until confluent state at 37 °C in a humidified atmosphere of 5% and then incubated with p(NIPAM)-co-5%AA-co-FA-co-Dox (final Doxorubicin concentration of 20 µM) for 15 min, 30 min, 1 h, 2 h, 4 h, 6 h, 8 h, and 24 h. Untreated cells were used as the negative control for background fluorescence. Subsequently, the samples were washed with PBS without Ca^2+^ and Mg^2+^, detached by Trypsin-EDTA 1× (Sigma-Aldrich) treatment and collected by centrifugation at 1000 rpm for 5′. The pellets were re-suspended in 500μL of PBS and analyzed by FACS-Canto cytometer (Germany) detecting the red (Dox) fluorescence emission (585 nm). For each sample were collected 1 × 10^5^ events investigated by BD FACS Diva software.

## Figures and Tables

**Table 1:** FTIR peaks of p(NIPAM), p(NIPAM)-co-5%AA, p(NIPAM)-co-5%AA-co-FA, and p(NIPAM)-co-5%AA-co-FA-co-Dox and their assignments with references.

Polymer	Peak No.	Peak (cm^−1^)	Bond Type	Reference
p(NIPAM)	1	3279	secondary amine	[38]
	2	2978	CH_3_ asymmetric stretch	[38]
	3	2938	CH_2_ asymmetric stretch	[38]
	4	2880	C-H stretch	[38]
	5	1625	amide I secondary	[39]
	6	1551	amide II	[39]
	7	1462	CH_2_ bend	[38,39]
	8	1389	CH_3_ bend	[38,39]
	9	1171	C-N stretch secondary amine	[38]
	10	1131	sulfate ion	[38]
p(NIPAM)-co-5%AA	11	3417	O-H group	[38,39]
	12	3283	secondary NH	[38,39]
	13	2972	CH_3_ asymmetric stretch	[38]
	14	2933	CH_2_ assymetric stretch	[38]
	15	2876	C-H stretch	[38]
	16	1632	amide I secondary	[39]
	17	1538	amide II	[39]
	18	1457	C-H2 bend	[38,39]
	19	1386	C-H3 bend	[38,39]
	20	1367	Carboxylate	[38]
	21	1171	C-N stretch secondary amine	[38]
	22	1130	sulfate ion	[38]
p(NIPAM)-co-5%AA-co-FA	23	3250	secondary amine	[38]
	24	2972	CH_3_ asymmetric stretch	[38]
	25	1635	amide I secondary	[39]
	26	1603	aromatic ring stretch	[38]
	27	1534	amide II	[39]
	28	1487	aryl C=C	[39]
	29	1387	Carboxylate group	[38]
	30	1339	aromatic secondary amine C-N	[38]
	31	1173	C-N secondary amine	[38]
	32	1058	sulfate ion	[38]
p(NIPAM)-co-5%AA-co-FA-co-Dox	33	3251	secondary amine	[38]
	34	2969	CH_3_ asymmetric stretch	[38]
	35	2938	CH_2_ asymmetric stretch	[38]
	36	1635	amide I secondary	[39]
	37	1604	aromatic ring stretch	[38]
	38	1531	amide II	[39]
	39	1487	aryl C=C	[39]
	40	1377	C13-H	[37]
	41	1342	aromatic secondary amine C-N	[38]
	42	1209	COH of Dox	[37]
	43	1171	C-N secondary amine	[38]
	44	1047	sulphate ion	[38]

## Supplementary Materials

The following are available online at https://www.mdpi.com/article/10.3390/gels7040203/s1, Figure S1: Folic acid calibration standard curve and calculation of folate conjugated to p(NIPAM)-co-5%AA [p(NIPAM)-co-5%AA-co-FA]; Figure S2: Doxorubicin calibration standard curve and calculation of drug conjugated to p(NIPAM)-co-5%AA-co-FA [p(NIPAM)-co-5%AA-co-FA-co-Dox]; Figure S3: Cooling cycles of p(NIPAM)-co-5%AA, p(NIPAM)-co-5%AA-co-FA and p(NIPAM)-co-5%AA-co-FA-co-Dox in contrast with cooling cycles shown in Figure 2; Figure S4: Cooling cycles of p(NIPAM)-co-5%AA, p(NIPAM)-co-5%AA-co-FA and p(NIPAM)-co-5%AA-co-FA-co-Dox in respect to their electrophoretic mobility in contrast with their heating cycles shown in Figure 3; Figure S5: Cell viability of HB 2 and MDA-MB 231 cells treated with 0; 12.5; 25; 50 and 100 µg/mL of p(NIPAM)-co-5%AA for 24 h (a) and 48 h (b). Cells incubated with doxorubicin were used as positive control; Figure S6: Acridine orange assay on MDA-MB 231 cells treated with 12.5 (c) or 100 µg/mL (d) of p(NIPAM)-co-5%AA for 24 h. Untreated cells and cells incubated with doxorubicin were used as negative (a) and positive (b) control, respectively; Figure S7: Confocal microscopy of MDA-MB 231 cells treated with 100 µg/mL of p(NIPAM)-co-5%AA-co-LY for 15′ (a–a’’); 30′ (b–b’’); 1 h (c–c’’); 2 h (d–d’’); 4 h (e–e’’); 6 h (f–f’’); 8 h (g–g’’) and 24 h (h–h’’). Red: bromide ethidium (DNA and RNA). Green: p(NIPAM)-co-5%AA-co-LY. Magnification 60×; Figure S8: Confocal microscopy of MDA-MB 231 cells treated for 1 h with 100 µg/mL of p(NIPAM)-co-5%AA-co-LY. Red: bromide ethidium (DNA and RNA). Green: p(NIPAM)-co-5%AA-co-LY. Magnification 160×; Figure S9: Fluorescence images of co-culture of HB2 (blue) and MDA-MB231 (blue and green) cells incubated with Doxorubicin (10 µM) (red) for 30 min (a–d); 1 h (a’–d’); 2 h (a’’–d’’) and 4 h (a’’’–d’’’). Blue: nuclei (DAPI); Green: MDA-MB 231 cells (CellTrace CFSE); Red: doxorubicin. Magnification 40×; Figure S10: Cytograms of flow cytometric analysis of HB2 and MDA-MB 231 cells incubated with 25 µM of doxorubicin conjugated to microgel (p(NIPAM)-co-5%AA-co-FA-co-Dox).
