# Supplementary material for: Functionalized Poly(N-isopropylacrylamide)-Based Microgels in Tumor Targeting and Drug Delivery
Source: Gels. 2021 Nov 8;7(4):203. doi: 10.3390/gels7040203 (PMC8628686; doi:10.3390/gels7040203)
Supplement: Supplementary file 1 [file gels-07-00203-s001.zip › gels-1431307-supplementary.pdf]

Supplementary Materials

Functionalized Poly(*N*-isopropylacrylamide)-Based Microgels in Tumor Targeting and Drug Delivery

Simona Campora [1,2,†], Reham Mohsen [3,4,†], Daniel Passaro [1], Howida Samir [3], Hesham Ashraf [3], Saif El-Din Al-Mofty [5], Ayman A. Diab [3], Ibrahim M. El-Sherbiny [5], Martin J. Snowden[4] and Giulio Ghersi [1,2,*]

[1] Department of Biological, Chemical and Pharmaceutical Sciences and Technologies [STEBICEF], University of Palermo, Viale delle Scienze, Ed. 16, 90128 Palermo, Italy; simona.campora@unipa.it (S.C.); daniel.passaro@abbvie.com (D.P.)

[2] Abiel s.r.l, c/o Arca Incubatore di Imprese , University of Palermo, Viale delle Scienze, Ed. 16 (Floor-2), 90128 Palermo, Italy

[3] Faculty of Biotechnology, October University for Modern Sciences and Arts, Cairo 12451, Egypt; rmohsen@msa.edu.eg (R.M.); ho.samir@nu.edu.eg (H.S.); hesham.ashraf@msa.edu.eg (H.A.); adiab@msa.eun.eg (A.A.D.)

[4] School of Science, University of Greenwich, Chatham, Kent, Canterbury ME4 4TB, UK; m.j.snowden@greenwich.ac.Gillingham

[5] Center of Materials Science, Zewail City of Science and Technology, 6th October City, Giza 12588, Egypt; s-saifel-din.el-mofty@zewailcity.edu.eg (S.E.-D.A.-M.); ielsherbiny@zewailcity.edu.eg (I.M.E.-S.)

* Correspondence: giulio.ghersi@unipa.it

† These authors contributed equally to this work.

1. Materials and methods

1.1. Synthesis of p(NIPAM)-co-5%AA-co-LY microgel

In a 1 L reaction vessel, 0.5 g of the initiator (potassium per sulphate (KPS)) was dissolved in 800 mL of distilled water. A three-necked lid was clamped to the reaction vessel, which was then heated to 70°C with continuous stirring. Monomer (4.5 g NIPAM), co-monomers (0.25 g Acrylic acid (AA) and 0.25 g Lucifer yellow (LY)) and cross linker (0.5g *N,N′*-methylenebisacrylamide) were stirred in distilled water (200 mL) and transferred into the reaction vessel containing the initiator and continuously stirred at 70°C for six hours under an inert atmosphere of nitrogen. When the reaction was complete, the nanogel dispersion was allowed to cool to room temperature. The nanogel was dialyzed in fresh de-ionised water for a week (changed daily), centrifuged and freeze-dried.

1.2. Quantification of folic acid and doxorubicin conjugated to p(NIPAM)-co-5%AA

The amounts of folic acid or doxorubicin conjugated to p(NIPAM)-co-5%AA were calculated by a spectrophotometric assay. Therefore, the samples [p(NIPAM)-co-5%AA-co-FA and p(NIPAM)-co-5%AA-co-FA-co-Dox] absorbances were determined at the wavelengths of 340 nm for folate and 485 nm for doxorubicin by Synergy™ HT Multidetection microplate reader spectrophotometer (BioTek). Different standard curves were performed using different concentrations of folic acid (0.05; 0.1; 0.15; 0.2; 0.25; 0.3; 0.35; 0.4; 0.45 and 0.5 mg/mL) or doxorubicin (5; 10; 20; 40; 60; 80 and 100 µM). In both cases, to set the machine p(NIPAM)-co-5%AA was used.

1.3. Viability of cells treated with microgels

Viability assay was performed on MDA-MB 231 or HB2 cells incubated with p(NIPAM)-co-5%AA microgels (Biocompatible assay). Cells were seeded on 96-well plates at the density of 1 x 10^4 cells/well and grown in the opportune medium at 37°C overnight. Therefore, cells were treated with p(NIPAM)-co-5%AA (12.5; 25; 50; 100 µg/mL) for 24h or 48h and cell viability was detected by using Cell Counting Kit-8 (CCK-8). WST-8 was added to each sample (1:10 dilution in complete medium) and incubated at 37°C for 2h. Spectrophotometric analysis at 450 nm was then performed to determine

the percentage of viable cells relative to the negative control (untreated cells). Cells treated with Doxorubicin were considered as a positive control.

1.4. Acridine Orange assay

Biocompatibility of p(NIPAM)-co-5%AA was tested by acridine orange assay on MDA-MB 231 cells. Cells were seeded into 12-well plates containing sterile coverslips at a density of 5 x 10^3 cells per well with complete D-MEM high glucose medium and grown for 24h at 37°C and for more 24h in presence of 12.5 or 100 µg/mL of p(NIPAM)-co-5%AA. Doxorubicin (5 µM) was used as an apoptotic inducer (positive control). After washing with PBS, cells were treated with 100 µg/mL of Acridine Orange/Ethidium Bromide solution (AO/EtBr) for 10 minutes at room temperature and analysed by fluorescence microscopy: Acridine orange is a dye that emits green fluorescence when bound to dsDNA and red fluorescence when bound to ssDNA or RNA.

1.5. Qualitative uptake by confocal microscopy

MDA-MB 231 cells were grown for 24 h at a density of 5X10^3 cells per well into 12 well culture plates containing a sterile glass coverslip in complete medium, at 37 °C and 5% CO_2. Following, the cells were incubated with 100 µg/mL of p(NIPAM)-co-AA-co-LY microgel for different times (15'; 30'; 1h; 2h; 4h; 6h and 24h) and then washed with PBS to remove the conjugates that were not taken up by the cells. After, they were fixed with 3.7 % formaldehyde (w/v) for 15 minutes and nuclei were labelled with DAPI (dilution of 1: 10000 in water) for 15 minutes at room temperature. Therefore, samples were detected by confocal microscopy (FLUOVIEW FV10i-LIV, Olympus). Untreated cells were used to calibrate the background fluorescence.

2. Results

2.1. Determination of folic acid or doxorubicin conjugated to p(NIPAM)-co-5%AA

The amount of folic acid conjugated to p(NIPAM)-co-5%AA was calculated by spectrophotometric analysis, reading the samples at 340 nm (folate peak). The amount of 0.462 mg/mL of small molecules linked was evaluated on p(NIPAM)-co-5%AA-co-FA using the equation of the straight line (y= 6.8078x) generated by a standard calibration curve (Figure S1). Similarly, the amount of conjugated doxorubicin was evaluated to be 68.4 µg/mL (Figure S2).

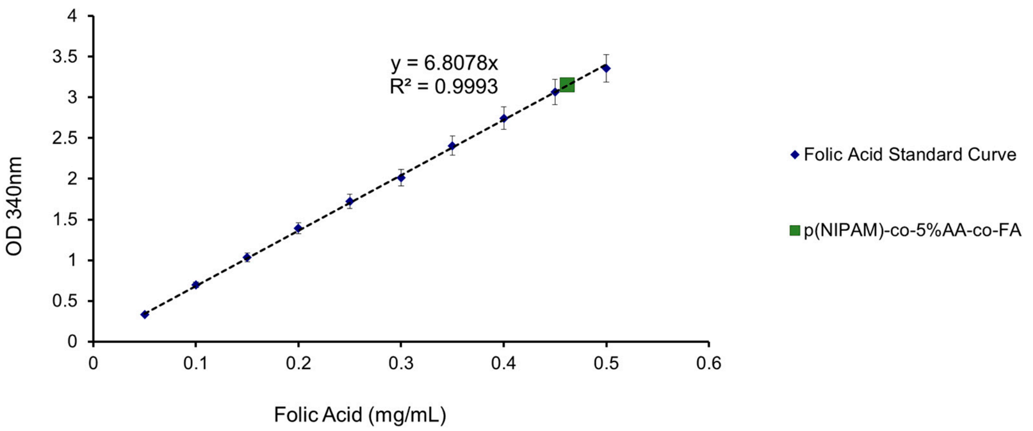

Figure S1. Folic acid calibration standard curve and calculation of folate conjugated to p(NIPAM)-co-5%AA [p(NIPAM)-co-5%AA-co-FA].

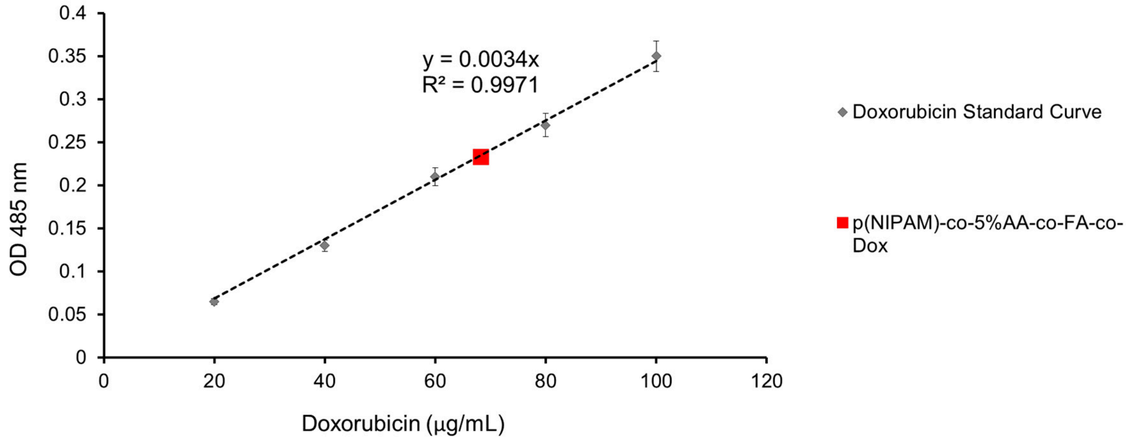

Figure S2. Doxorubicin calibration standard curve and calculation of drug conjugated to p(NIPAM)-co-5%AA-co-FA [p(NIPAM)-co-5%AA-co-FA-co-Dox]. Diliution factor: 3.

2.2. Size and zeta potential of the microgels (Cooling cycles)

Figures S3 and S4 show the cooling cycles of p(NIPAM)-co-5%AA, p(NIPAM)-co-5%AA-co-FA and p(NIPAM)-co-5%AA-co-FA-co-Dox showing reversibility of their swelling/deswelling behaviour in terms of size (Figure S3) and electrophoretic mobility (E_m; Figure S4) [1,2]. The PDI in the cooling cycles are 0.107, 0.482 and 0.531 for p(NIPAM)-co-5%AA, p(NIPAM)-co-5%AA-co-FA, and p(NIPAM)-co-5%AA-co-FA-co-Dox respectively.

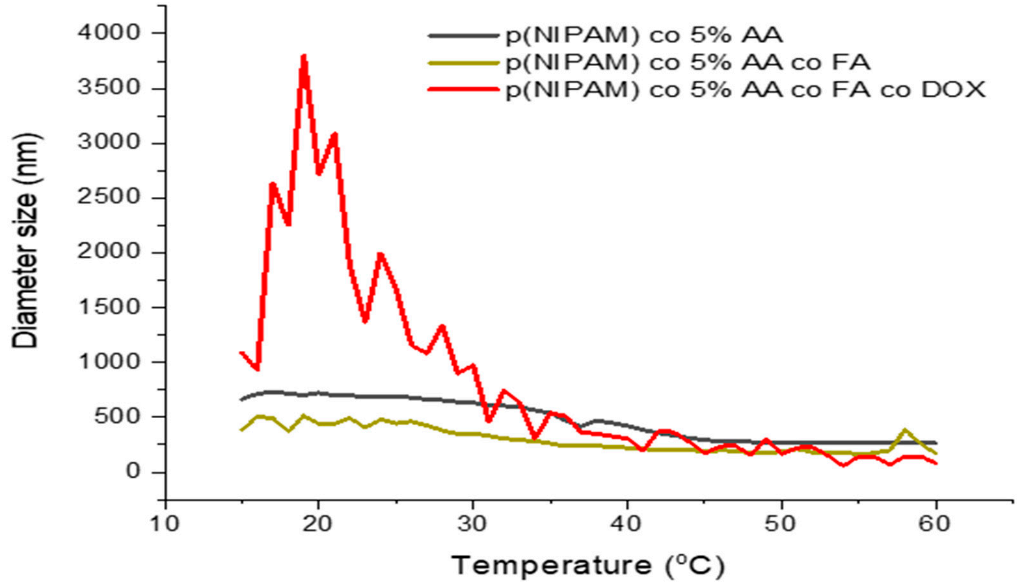

Figure S3. Cooling cycles of p(NIPAM)-co-5%AA, p(NIPAM)-co-5%AA-co-FA and p(NIPAM)-co-5%AA-co-FA-co-Dox in contrast with cooling cycles shown in fig.2.

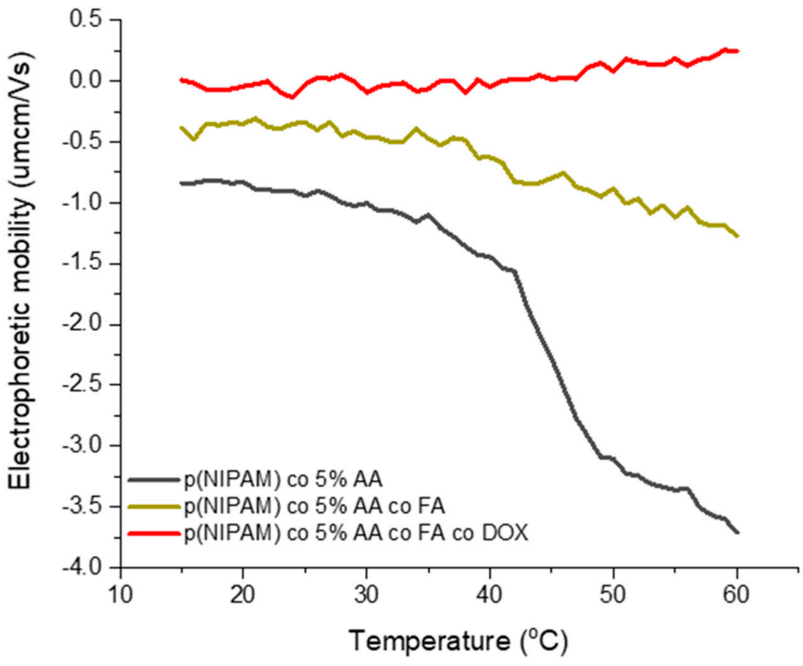

Figure S4. Cooling cycles of p(NIPAM)-co-5%AA, p(NIPAM)-co-5%AA-co-FA and p(NIPAM)-co-5%AA-co-FA-co-Dox in respect to their electrophoretic mobility in contrast with their heating cycles shown in figure 3.

2.3. Cell biocompatibility

Biocompatibility of p(NIPAM)-co-5%AA was performed on healthy HB2 and tumour MDA-MB231 cells incubated with different microgel concentrations for 24 and 48h (Figure S5).

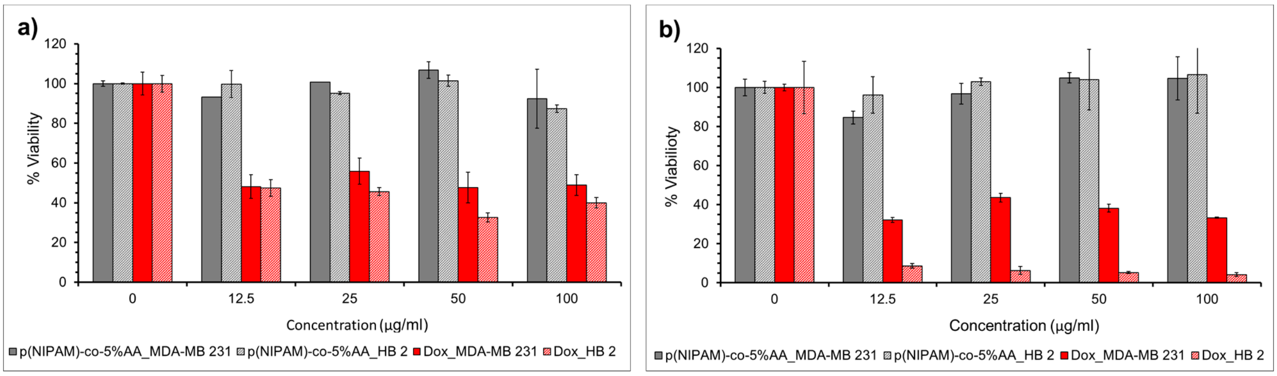

Figure S5. Cell viability of HB 2 and MDA-MB 231 cells treated with 0; 12.5; 25; 50 and 100 µg/mL of p(NIPAM)-co-5%AA for 24h (a) and 48 h (b). Cells incubated with doxorubicin were used as positive control.

The cell viability was maintained around 100% even at highest p(NIPAM)-co-5%AA concentration (100 µg/mL) and for longer time (48h), suggesting good biocompatibility that was further investigated by acridine orange assay (Figure S6).

MDA-MB 231 cells were incubated with 12.5 and 100 mg/mL (Figure S6 c and d) of p(NIPAM)-co-5%AA for 24h. As shown in figure S6, cells present green fluorescence as the negative control (untreated cells, figure S6 a), due to the bind of acridine orange stain to dsDNA, typical of alive cells. On the contrary, the positive control (cells treated with 5µM of doxorubicin, figure S6 b) showed the red fluorescence due to the bind with ssDNA (dead cells).

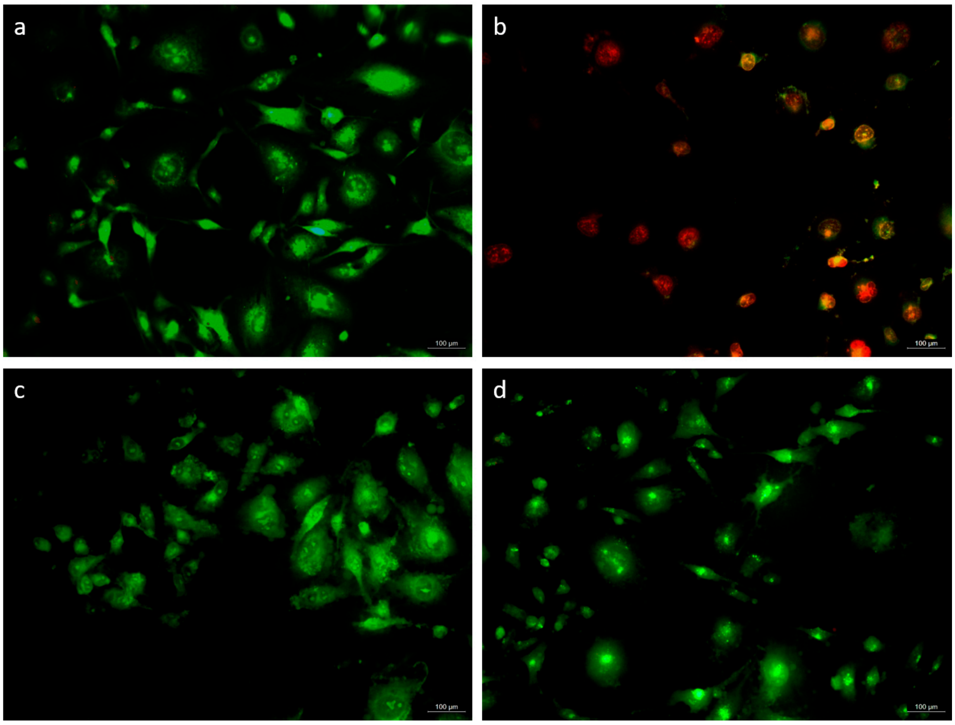

Figure S6. Acridine orange assay on MDA-MB 231 cells treated with 12.5 (c) or 100 μg/mL (d) of p(NIPAM)-co-5%AA for 24h. Untreated cells and cells incubated with doxorubicin were used as negative (a) and positive (b) control, respectively.

2.4. p(NIPAM)-co-5%AA-co-LY cellular uptake

Microgel cellular uptake was investigated by treating MDA-MB231 cells with microgels conjugated to the green fluoresce probe Lucifer yellow (p(NIPAM)-co-5%AA-co-LY) over time. The microgels started to migrate into the cell at 15 minutes (Figure S7 a-a'') and their concentration increase inside cells in a time-dependent manner. After 1 hour of incubation, green fluorescence relative to p(NIPAM)-co-5%AA-co-LY appeared less diffused in the cytoplasm and more localized in specific areas, probably corresponding to the Golgi apparatus or the endoplasmic reticulum (Figure S7 c-c'' and S8). Specific localization was maintained for longer incubation times (2h; 4h, 6h, figure S7 d-d'', e-e'', f-f'') and the green fluorescence appeared decreased after 8h and more after 24h of incubation.

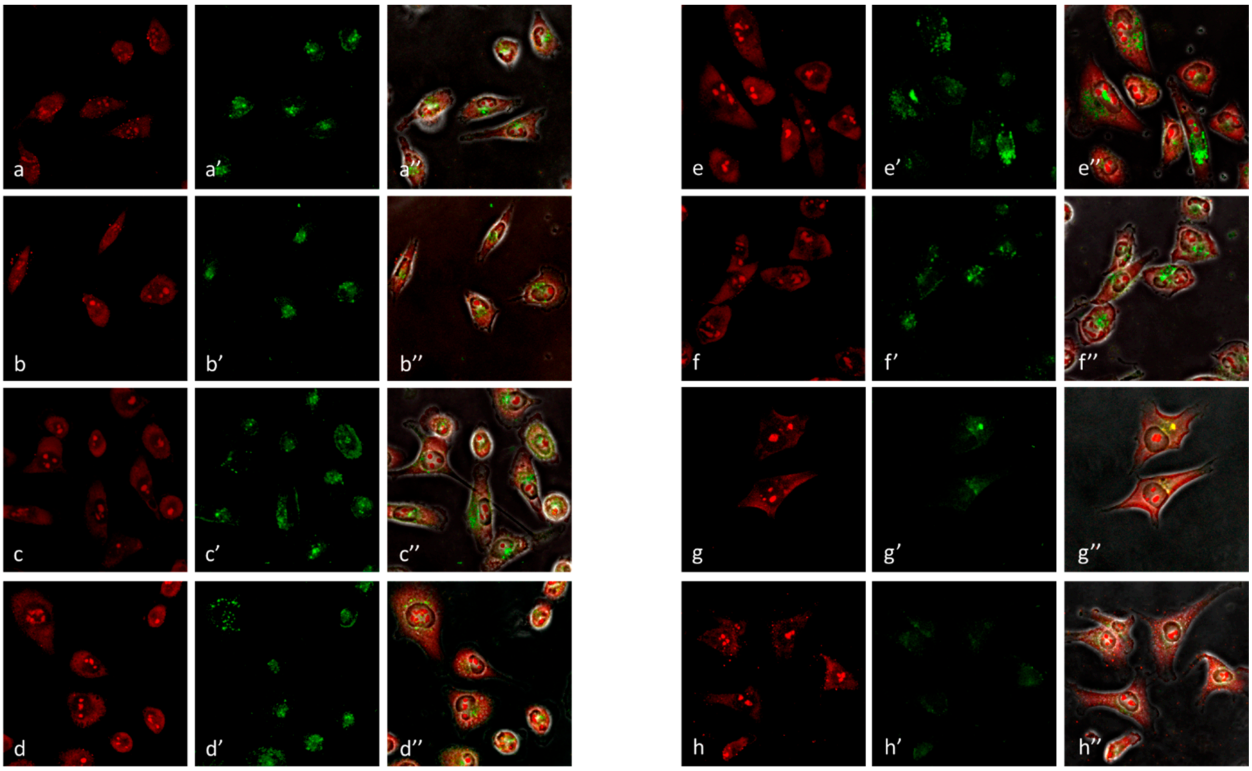

Figure S7. Confocal microscopy of MDA-MB 231 cells treated with 100 µg/mL of p(NIPAM)-co-5%AA-co-LY for 15′ (a-a′′); 30′ (b-b′′); 1h (c-c′′); 2h (d-d′′); 4h (e-e′′); 6h (f-f′′); 8h (g-g′′) and 24h (h-h′′). Red: bromide ethidium (DNA and RNA). Green: p(NIPAM)-co-5%AA-co-LY. Magnification 60X.

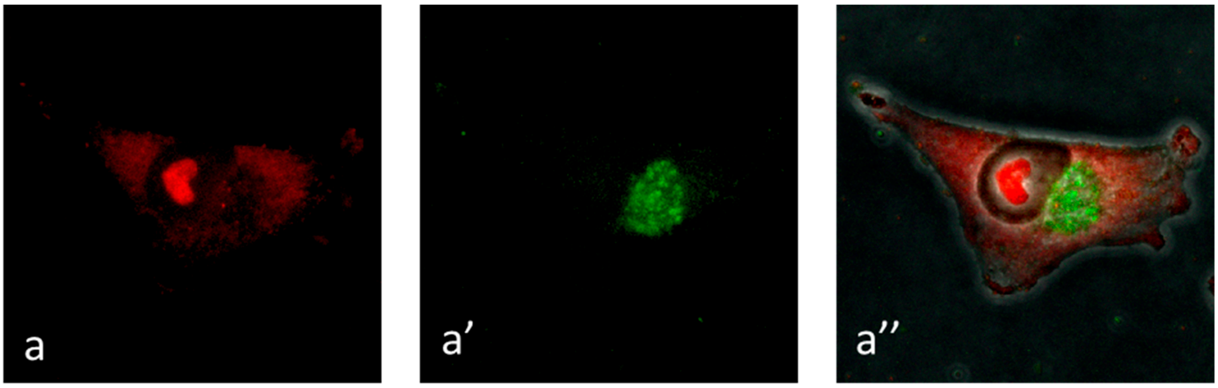

Figure S8. Confocal microscopy of MDA-MB 231 cells treated for 1 h with 100 µg/mL of p(NIPAM)-co-5%AA-co-LY. Red: bromide ethidium (DNA and RNA). Green: p(NIPAM)-co-5%AA-co-LY. Magnification 160X.

2.5. Qualitative uptake of Dox in co-culture experiment

Co-culture experiment was performed as reported in the paper to follow the specific cell tumour uptake of p(NIPAM)-co-5%AA-co-FA-co-Dox. As a control, the same experiment was performed by incubating cells with the same amount of soluble form doxorubicin (10 µM) for the identical times (30′, 1h 2h and 4h, figure S9). Red fluorescence relative to doxorubicin, appear inside cells just after 30′ of incubation, suggesting that it can enter both in normal (HB2) and tumour (MDA-MB 231) cells.

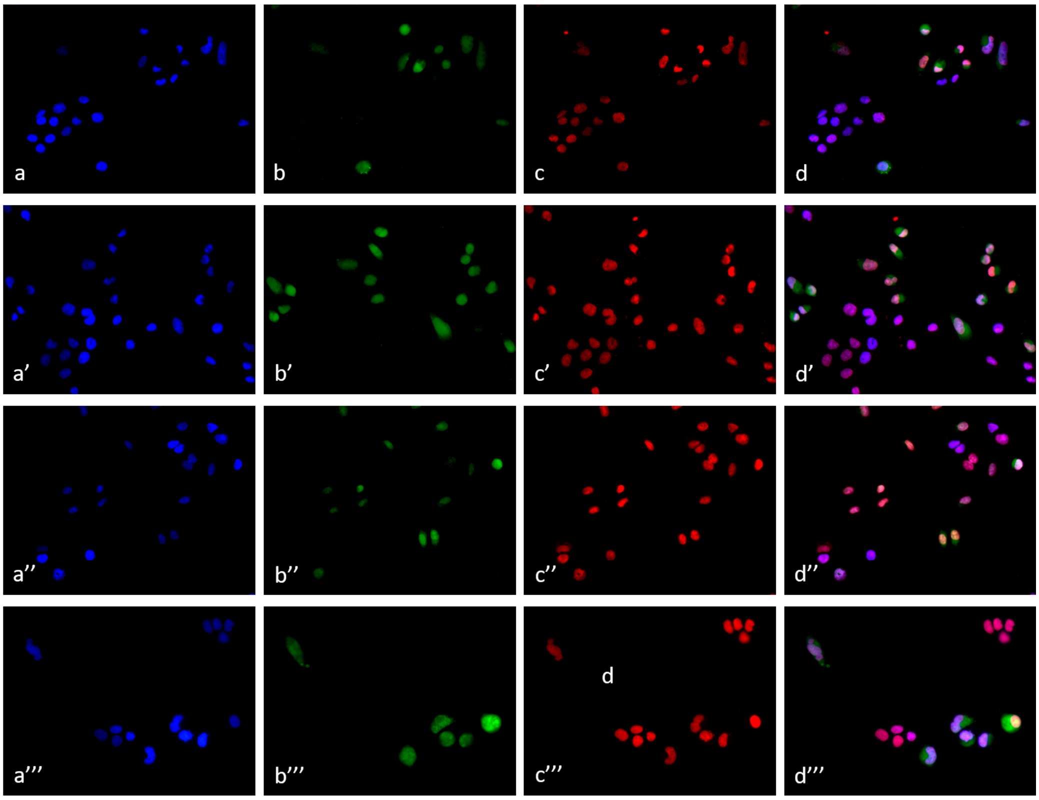

Figure S9. Fluorescence images of co-culture of HB2 (blue) and MDA-MB231 (blue and green) cells incubated with Doxorubicin (10 μM) (red) for 30 minutes (a-d); 1 hour (a′-d′); 2 hours (a′′-d′′) and 4 hours (a′′′-d′′′). Blue: nuclei (DAPI); Green: MDA-MB 231 cells (CellTrace CFSE); Red: doxorubicin. Magnification 40X.

2.6. Quantitative microgel cellular uptake

Quantitative microgel cellular uptake was evaluated by flow cytometric analysis of both HB2 and MDA-MB231 cells incubated with p(NIPAM)-co-5%AA-co-FA-co-Dox for 15′, 30′, 1h, 2h, 4h, 6h, 8h and 24h as described in the paper. Cytograms are reported in figure S10 and show the time-dependent microgel uptake and the higher microgel internalization into cancer MDA-MB 231 cells.

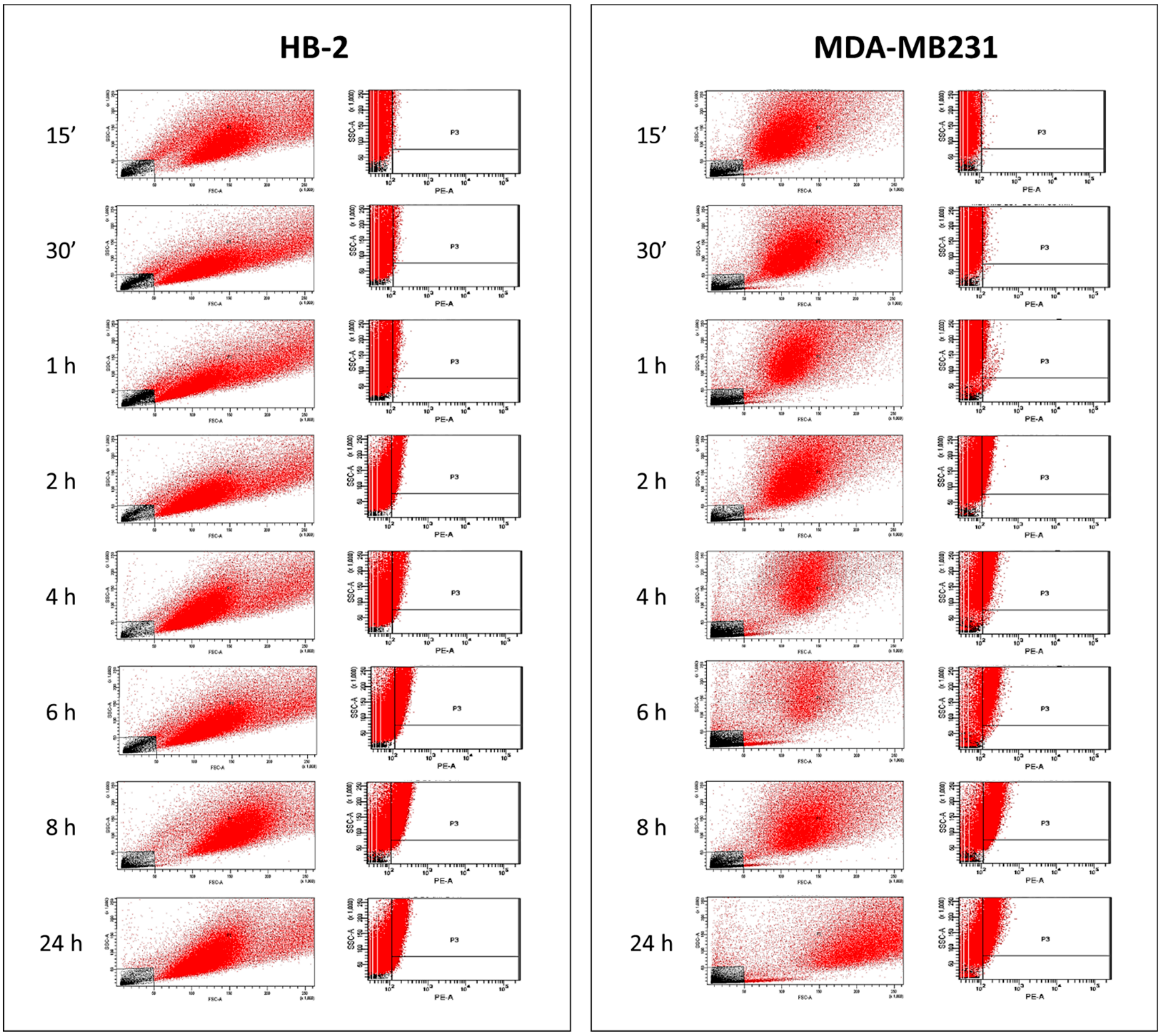

Figure S10. Cytograms of flow cytometric analysis of HB2 and MDA-MB 231 cells incubated with 20 μM of doxorubicin conjugated to microgel (p(NIPAM)-co-5%AA-co-FA-co-Dox).

References

1. Dowding, P.J.; Vincent, B.; Williams, E. Preparation and swelling properties of poly(NIPAM) "minigel" particles prepared by inverse suspension polymerization. *J. Colloid Interface Sci.* **2000**, *221*, 268–272, doi:10.1006/jcis.1999.6593.
2. Farooqi, Z.H.; Khan, H.U.; Shah, S.M.; Siddiq, M. Stability of poly(N-isopropylacrylamide-co-acrylic acid) polymer microgels under various conditions of temperature, pH and salt concentration. *Arab. J. Chem.* **2017**, *10*, 329–335, doi:10.1016/j.arabjc.2013.07.031.
